# Identification of eQTLs for Hepatic *Xbp1s* and *Socs3* Gene Expression in Mice Fed a High-Fat, High-Caloric Diet

**DOI:** 10.1534/g3.115.016626

**Published:** 2015-01-23

**Authors:** Sarina Pasricha, Jane Kenney-Hunt, Kristy Anderson, Nadereh Jafari, Rabea A. Hall, Frank Lammert, James Cheverud, Richard M. Green

**Affiliations:** * Department of Internal Medicine, Northwestern University, Chicago, Illinois; † Department of Internal Medicine, University of North Carolina, Chapel Hill, North Carolina; ‡ Department of Biology and Environmental Science, Westminster College, Fulton, Missouri; § Center for Genetic Medicine, Northwestern University, Chicago, Illinois; ** Department of Medicine II, Saarland University Medical Center, Homburg, Germany; †† Department of Biology, Loyola University, Chicago, Illinois

**Keywords:** eQTLs, mice, fatty, liver

## Abstract

Nonalcoholic fatty liver disease (NAFLD) is a highly prevalent form of human hepatic disease and feeding mice a high-fat, high-caloric (HFHC) diet is a standard model of NAFLD. To better understand the genetic basis of NAFLD, we conducted an expression quantitative trait locus (eQTL) analysis of mice fed a HFHC diet. Two-hundred sixty-five (A/J × C57BL/6J) F_2_ male mice were fed a HFHC diet for 8 wk. eQTL analysis was utilized to identify genomic regions that regulate hepatic gene expression of *Xbp1s* and *Socs3*. We identified two overlapping loci for *Xbp1s* and *Socs3* on Chr 1 (164.0–185.4 Mb and 174.4–190.5 Mb, respectively) and Chr 11 (41.1–73.1 Mb and 44.0–68.6 Mb, respectively), and an additional locus for *Socs3* on Chr 12 (109.9–117.4 Mb). C57BL/6J-Chr 11^A/J^/ NaJ mice fed a HFHC diet manifested the A/J phenotype of increased *Xbp1s* and *Socs3* gene expression (*P* < 0.05), whereas C57BL/6J-Chr 1^A/J^/ NaJ mice retained the C57BL/6J phenotype. In addition, we replicated the eQTLs on Chr 1 and Chr 12 (LOD scores ≥3.5) using mice from the BXD murine reference panel challenged with CCl_4_ to induce chronic liver injury and fibrosis. We have identified overlapping eQTLs for *Xbp1* and *Socs3* on Chr 1 and Chr 11, and consomic mice confirmed that replacing the C57BL/6J Chr 11 with the A/J Chr 11 resulted in an A/J phenotype for *Xbp1* and *Socs3* gene expression. Identification of the genes for these eQTLs will lead to a better understanding of the genetic factors responsible for NAFLD and potentially other hepatic diseases.

Nonalcoholic fatty liver disease (NAFLD) is the most common cause of abnormal liver function tests and is one of the most frequent causes of chronic liver disease in the United States. NAFLD has a prevalence as high as 30% in the general population and 75% in obese patients (Lazo and Clark 2008; Reid 2001; Wanless and Lentz 1990; Browning and Horton 2004). NAFLD represents a spectrum of diseases, including simple steatosis, nonalcoholic steatohepatitis (NASH) with or without fibrosis, cirrhosis, and end-stage liver disease. NAFLD has been closely linked to the metabolic syndrome characterized by obesity, dyslipidemia, and insulin resistance (Ron and Walter 2007; Vanni *et al.* 2010; Speliotes *et al.* 2008; Speliotes *et al.* 2010). Liver-related and overall mortality rates are significantly higher in patients with NAFLD compared with the general population (Ong *et al.* 2008). In fact, it is estimated that NAFLD/cryptogenic cirrhosis will be the leading indication for liver transplantation within a decade. Despite the high prevalence of NAFLD, the pathogenesis and genetic mechanisms responsible for the susceptibility and progression of NAFLD remain poorly understood.

Several studies support the important role of genetics in the pathogenesis of nonalcoholic steatohepatitis (Willner *et al.* 2001; Angulo 2002; Silverman *et al.* 1989; Wagenknecht *et al.* 2009). NASH is a polygenic disease in which susceptible individuals can develop disease when exposed to environmental factors such as excess calorie intake, obesity, and insulin resistance (Speliotes *et al.* 2008; Willner *et al.* 2001; Angulo 2002). The unfolded protein response (UPR) is a protective cellular signaling response that is activated in response to endoplasmic reticulum (ER) stress, and the hepatic UPR is activated by insulin resistance, obesity, and fatty liver (Cnop *et al.* 2012; Eizirik *et al.* 2008; Ji and Kaplowitz 2006; Brenner *et al.* 2013). In fact, X-box binding protein-1 spliced (*Xbp1s*) is a key regulator of the UPR and dysregulation of hepatic *Xbp1s* has been shown to be important in the pathogenesis of human nonalcoholic fatty liver diseases (Puri *et al.* 2008). *Xbp1s* is the spliced form of *Xbp1* that occurs when the *Xbp1* transcript is spliced by Inositol Requiring Kinase 1-α (IRE1α) in response to ER stress. When this occurs, *Xbp1s* regulates the expression of its down-stream target genes (Ron and Walter 2007; Puri *et al.* 2008; Kaser *et al.* 2008; Tilg and Moschen 2010; Lee *et al.* 2008).

Suppressor of Cytokine Signaling 3 (*SOCS3*) is an inflammatory mediator that has also been previously identified to be important in glucose and lipid metabolism, and it has been implicated in the pathogenesis of experimental models of NAFLD (Wang *et al.* 2009; Ihle 1995; Handy *et al.* 2011; Brenner *et al.* 2013; Moschen *et al.* 2010; Clementi *et al.* 2009; Ogawa and Kasuga 2008; Ueki *et al.* 2005; Tilg 2010). In fact, when mice with a liver-specific deletion of *Socs3* are fed a high-fat diet, they develop increased liver fat and inflammation compared with control mice (Sachithanandan *et al.* 2010). Therefore, both human and murine studies demonstrate that hepatic *XBP1s* and *SOCS3* expression are important in the pathogenesis of fatty liver diseases.

Quantitative trait loci (QTL) analysis is a well-established genetic technique for mapping the chromosomal loci of complex traits in rodents, humans, multiple animals, and plants (Rapp 2000; Paterson *et al.* 1991; Fullerton *et al.* 2003). QTL analysis using experimental crosses of mice has been successfully applied to identify QTL in several polygenic diseases of the metabolic syndrome, including obesity, diabetes, and hypertension (Mu *et al.* 1999; Pomp 1997; Wang *et al.* 2012). The aim of this investigation is to utilize the high-fat, high-caloric (HFHC) dietary model of murine fatty liver disease and eQTL analysis in an F_2_ intercross of C57BL/6J and A/J mice to identify eQTLs and candidate genes that are important in the pathogenesis and progression of NAFLD. In addition, we tested allele effects of candidate regions in consomic strains (C57BL/6J-Chr 1^A/J^/ NaJ and C57BL/6J-Chr 11^A/J^/ NaJ) where the chromosome carrying the eQTL region has been substituted from the A/J background into a C57BL/6J background.

To help determine the relevance of the identified loci in other forms of liver injury, we used the CCl_4_ hepatotoxicity model and the BXD murine reference panel for systems genetics (Andreux *et al.* 2012). BXD mice are recombinant inbred lines generated from F_1_ hybrids of C57BL/6J and DBA/2J mice by consecutive brother–sister matings of the F_2_ progeny for more than 20 generations. These genetically mosaic lines are homozygous at every genetic locus and represent a panel that has been extensively genotyped across the genomes.

Identifying candidate NAFLD genes will be essential for enhancing our understanding of the genetic factors that are important in the pathogenesis and progression of NAFLD and in subsequently developing rational therapeutic strategies for this common liver disease.

## Materials and Methods

### Animals

A/J, C57BL/6J, B6AF1/J, C57BL/6J-Chr 1^A/J^/ NaJ, and C57BL/6J-Chr 11^A/J^/ NaJ male mice were purchased from Jackson Laboratory (Bar Harbor, ME). A/J and C57BL/6J mice were chosen following a preliminary screen of six mouse strains (A/J, C57BL/6J, DBA-2J, AKR/J, 129x1/SvJ) for hepatic expression of *Xbp1s* and *Socs3*. The A/J and C57BL/6J recombinant inbred (RI) strains of mice had no significant differences in baseline hepatic *Xbp1s* and *Socs3* expression when fed chow, yet elicited different susceptibilities of hepatic *Xbp1s* and *Socs3* gene expression when fed the high-fat diet. C57BL/6J-Chr 1^A/J^/ NaJ mice are chromosome substitution strains (CSS) of consomic mice where chromosome 1 from strain A/J has been substituted on a C57BL/6J background; C57BL/6J-Chr 11^A/J^/ NaJ mice are CSS mice where chromosome 11 from strain A/J has been substituted into a C57BL/6J background. Chromosomes 1 and 11 substitute lines were selected because eQTLs for both *Xbp1s* and *Socs3* localized to these chromosomes. B6AF1/J mice are an F_1_ hybrid of C57BL/6J female and A/J male mice, and these mice were crossed to generate 265 F_2_ generation mice from 74 litters that were used for eQTL analysis. Male 8- to 10-wk-old mice were used for all experiments because differences in hepatic *Xbp1s* and *Socs3* expression were slightly greater in male compared with female mice. We also analyzed 30 BXD recombinant inbred lines with an average of six mice per sex and line. Mice were purchased (BXD lines 1, 2, 6, 11, 12, 13, 14, 19, 24a, 27, 28, 31, 32, 33, 34, 39, 40, 42, 96, and 98) from Jackson Laboratory and (BXD lines 43, 51, 55, 62, 65, 69, 73, 75, 87, and 90) from Oak Ridge Laboratory. All animal protocols were approved by the Northwestern University Institutional Animal Care and Use Committee (IACUC) or according to all relevant welfare regulations of the Animal Care and Use Committee for Saarland University that approved the protocols (TV 10/2008).

### Phenotypic analysis of mice

In HFHC diet feeding studies, A/J, C57BL/6J, C57BL/6J-Chr 1^A/J^/ NaJ, or C57BL/6J-Chr 11^A/J^/ NaJ mice were given free access to water and a HFHC diet (60% fat, 20% protein, 20% carbohydrates; D12492; Research Diets, New Brunswick, NJ) for 8 wk. Body weight was recorded after 0, 4, and 8 wk of dietary feeding. After 8 wk, the mice were killed using CO_2_ narcosis after a 4-hr fast, and blood was collected by cardiac puncture. The liver and spleen were rapidly excised, rinsed in ice-cold saline, weighed, and snap-frozen in liquid nitrogen. Tails were clipped and snap-frozen in liquid nitrogen and stored at −80° until analyzed. To assess for liver injury and components of the metabolic syndrome, hepatic triglyceride levels were measured enzymatically on liver homogenate according to the manufacturer (Pointe Scientific, Canton MI); serum alanine aminotransferase (ALT) was measured using a spectrophotometric assay kit (Biotron, Hemet, CA). Serum glucose was measured by the glucose oxidase method using a colorimetric meter (Glucose Assay Kit). Serum insulin levels were determined using the Meso Scale Discovery system (Gaithersburg, MD). Quantitative insulin sensitivity check index (QUICKI) was calculated using 4-hr fasting insulin and glucose levels. In RT-PCR experiments, total RNA isolation and reverse-transcription were performed from liver using Trizol (Invitrogen) and qScript One-Step CYBR Green qRT-PCR (Quanta Biosciences). *Xbp1s* forward primers were sense 5′-AAGAACACGCTTGGGAATGG-3′ and *Xbp1s* reverse primers were anti-sense 5′-CTGCACCTGCTGCGGAC-3′. *Socs3* forward primers were sense 5′-**CCCTTGCAGTTCTAAGTTCAACA**-3′ and *Socs3* reverse primers were anti-sense 5′-**ACCTTTGACAAGCGGACTCTC**-3′. *Gapdh* was used as an internal control and was unchanged under the experimental condition. Studies were performed in A/J, C57BL/6J, C57BL/6J-Chr 1^A/J^/ NaJ, or C57BL/6J-Chr 11^A/J^/ NaJ and F_2_ (A/J × C57BL/6J) mice. Comparisons were performed using Student’s *t*-test or ANOVA, and the Tukey range test was performed as a post-hoc test.

### eQTL analysis of mice fed a high-fat, high-caloric diet

DNA was extracted from mouse tails and medium density linkage panel genotyping was performed at the Center of Genomic Studies at Northwestern University using Illumina Medium Density Microarray Chips (Illumina, San Diego, CA); 959 polymorphic SNPs were identified between C57BL/6J and the A/J parental strains, and genotyping and phenotyping for hepatic gene expression were performed on 265 F_2_ male mice. eQTL analysis was performed using R/qtl (Broman *et al.* 2003). Genome-wide 5% significance thresholds were generated in R/qtl using the scanone function and 1000 permutations. The LOD threshold for autosomes is 3.91 and is 2.87 for the X chromosome. Additional chromosome-wide 5% significance thresholds were generated using the method of Li and Ji (2005) (Chen and Storey 2006). The phenotypes of *Xbp1s*, *Socs3*, glucose, and insulin were log-transformed before analysis. SNP genomic positions obtained from Ensembl.org, build GRCm38.p3. Genes, predicted genes, and differential SNPs and nsSNPs were obtained from Mouse Genome Informatics Database (MGI 5.20). eQTL regions are defined as the 1.0 LOD-drop of both sides of the peak of the eQTL. The SNP marker closest to the 1.0 LOD-drop was utilized as the physical boundary of the interval. *Xbp1* is located at Chr11: 5,520,659–5,525,893; murine *Socs3* is at Chr11: 117,966,079–117,970,047 bp.

### Microarray analysis of mice fed a high-fat, high-caloric diet

For microarray studies analyzing the differentially expressed hepatic genes between A/J and C57BL/6J mice, analysis was performed using a minimum of three samples of hepatic mRNA, with each sample pooled from two mice (San Diego, CA). We utilized Illumina Mouse Ref 8 chips containing 25,697 probes of 18,708 genes. All samples met the Illumina standard quality control checks. Data quality checks were performed using the Bioconductor Lumi package for R statistical programming environment. The intensity distribution of the samples was examined by histogram and box plots. The data processing also includes a normalization procedure utilizing a quantile normalization method (Bolstad *et al.* 2003) to reduce the obscuring variation between microarrays, which might be introduced during the processes of sample preparation, manufacture, fluorescence labeling, hybridization, and/or scanning. Hierarchical clustering and principal component analysis were performed on the normalized signal data to assess the sample relationship and variability.

Using the criteria that a probe is considered not to be expressed if no probes’ background *P* value was less than 0.01, 13,060 out of 25,697 probes were kept for further differential gene expression analyses. A modified *t*-test using limma’s algorithm of fitting to linear models was used to obtain an adjusted *P* value, using the Benjamini-Hochberg method to estimate the false discovery rate. For each requested comparison, genes that showed a fold-change in expression of at least 1.5 and an adjusted *P* value of 0.05 or less were considered differentially expressed. The probes’ genes were identified using the lumiMouse DB package (Bolstad *et al.* 2003).

### eQTL analysis of BXD mice treated with carbon tetrachloride (CCl_4_)

Mice from C57BL/6J, DBA/2J, B6D2 F1 hybrids and 30 BXD recombinant inbred lines were phenotyped for hydroxyproline levels and collagen areas after induction of chronic liver injury and fibrosis using CCl_4_. CCl_4_ (0.7 mg/kg body weight, in a 1:1 mixture with mineral oil) was administered for 6 wk with two intraperitoneal injections per week. The mice do not develop fibrosis when fed a chow diet without the administration of CCl_4_. We measured hepatic collagen contents (hydroxyproline levels and collagen areas) as quantitative parameters and fibrosis stages as semiquantitative measures in histological liver sections as previously described (Hall *et al.* 2014). Phenotypic characterization of hepatic collagen was initially performed in male and female mice. Transcriptomes of CCl_4_-treated BXD lines were analyzed in female mice (three arrays per line) because they showed more pronounced differences in fibrosis progression among the BXD lines. The mice were chow-fed and the transcriptome of three female mice from each BXD line after CCl_4_ challenge was determined with Affymetrix Mouse Gene 1.0 ST microarrays. These data underwent quality control and normalization and were uploaded into the GeneNetwork database (http://www.genenetwork.org/webqtl/main.py). Using the gene expression values as quantitative traits, interval mapping was performed to identify eQTLs as implemented in GeneNetwork.

## Results

### Hepatic *Xbp1s* and *Socs3* gene expression in A/J and C57BL/6J mice fed a HFHC diet

**Figure 1:**
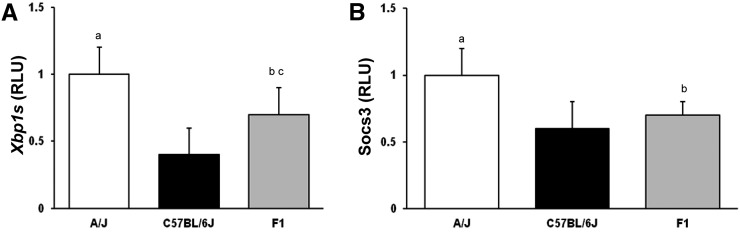
Hepatic mRNA expression of *Xbp1s* and *Socs3* in A/J, C57BL/6, and F1(A/J × C57BL/6J) mice. Mice were fed a 60% high-fat, high-caloric (HFHC) diet for 8 wk and hepatic mRNA expression of (A) *Xbp1s* and (B) *Socs3* measured using RT-PCR. RLU, random light units. Mean ± SEM. A/J (white), C57BL/6J (black), F1(A/J × C57BL/6J) (gray) mice; ap < 0.05 *vs.* C57BL/6J; bp < 0.05 *vs.* A/J mice; cp < 0.05 *vs.* C57BL/6J mice (n = 8 for all groups).

Parental mouse strains C57BL/6J and A/J fed a HFHC diet for 8 wk elicited significant differences in the hepatic gene expression of *Xbp1s*: 1.0 ± 0.2 *vs.* 0.4 ± 0.2 in A/J and C57BL/6J mice, respectively (*P* < 0.05). There was also a similar difference in hepatic *Socs3 gene* expression: 1.0 ± 0.2 *vs.* 0.6 ± 0.2 in A/J and C57BL/6J mice, respectively (*P* < 0.05). Hepatic *Xbp1s* and *Socs3* gene expression in F_1_ (A/J × C57BL/6J) mice was 0.7 ± 0.1 for *Xbp1s* and 0.7 ± 0.2 for *Socs3* (*P* < 0.05), both significantly different from A/J mice. Hepatic *Xbp1s* expression also differed between the F_1_ (A/J × C57BL/6J) and C57BL/6J mice (Figure 1; n = 8 for all groups). Supporting Information, Table S1 shows the Pearson correlation matrix of the phenotypes *Xbp1s* and *Socs3* in C57BL/6J and A/J mice (as well as for several phenotypes of the metabolic syndrome).

### Identification of eQTLs for hepatic *Xbp1s* and *Socs3* gene expression using F_2_ (A/J × C57BL/6J) mice fed a HFHC diet

**Figure 2:**
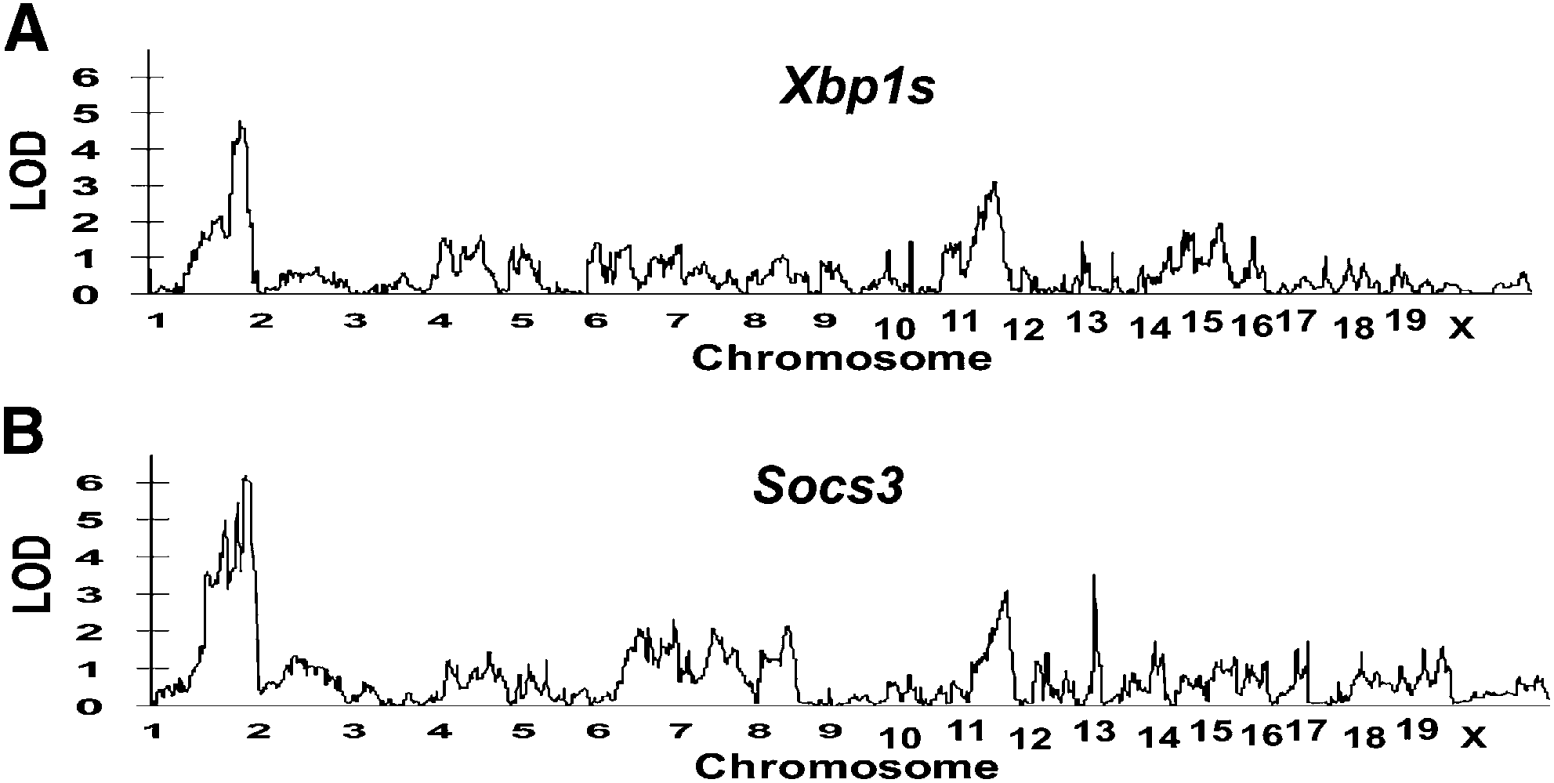
Whole genome LOD plots of eQTLs identified in A/J and C57BL/6J mice fed a high-fat diet.

**Figure 3:**
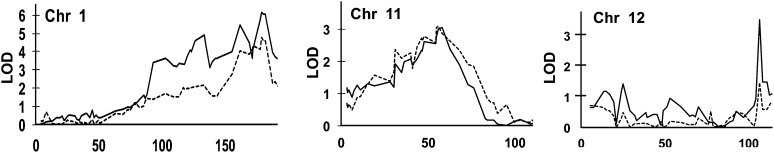
eQTL linkage maps of A/J and C57BL/6J mice fed a high-fat diet. *Xbp1s* (dashed line). *Socs3* (solid line).

*Xbp1* and *Socs3* are both located on mouse chromosome 11 (*Xbp1*: 5.5 Mb and *Socs3:* 118.0 Mb). We identified an overlapping eQTL for *Xbp1s* and *Socs3* on chromosome 1 (181.5 Mb), with LOD scores of 4.79 and 6.18, respectively. Additional overlapping eQTLs were also identified on chromosome 11 (56.2 and 58.4 Mb). There was also an eQTL identified for *Socs3* on chromosome 12 (113.4 Mb) with a LOD score of 3.53 (Table 1). Figure 2 and Figure 3 show the whole genome LOD plots and the eQTL linkage maps, respectively. Microarray analysis identified eight and nine differentially expressed candidate genes within the overlapping eQTLs on chromosomes 1 and 11, respectively; five differentially expressed genes were identified in the *Socs3* eQTL on chromosome 12 (Table 2).

**Table 1:** Mouse chromosome eQTL locations for *Xbp1s* and *Socs* using F_2_ (A/J × C57BL/6J) mice

CHR	Trait	LOD	SNP	Position (Mb)	Interval (Mb)	# Genes	Diff. SNPs	*a*	*a/SD*	*d*	*d/SD*	*d/a*
1	*Xbp1s*	4.79	rs 13476265	181.5	162.0–183.6	265	24,755	0.123	0.375	−0.051	−0.155	−0.413
1	*Socs3*	6.18	rs 13476265	181.5	172.5–188.7	151	14,519	0.129	0.407	−0.077	−0.244	−0.600
11	*Xbp1s*	3.11	rs 3684076	56.2	41.3–73.3	503	14,835	−0.101	−0.307	−0.054	−0.165	0.537
11	*Socs3*	3.09	rs 3697686	58.4	44.2–68.8	337	11,613	−0.100	−0.315	−0.046	60.463	0.463
12	*Socs3*	3.53	rs 13481655	113.4	108.7–116.2	79	6446	0.046	0.145	−0.131	−0.415	−2.865

Positive “a” values indicate that at a locus, the homozygous C57BL/6Jt trait mean is greater than the mean of the A/J homozygotes. Negative “d” values indicate that at that locus, the heterozygote trait mean is closer to the A/J homozygote mean than the C57BL/6J homozygote mean. CHR, chromosome; SNP, SNP marker closest to peak LOD score; Interval = 1.0–LOD support interval surrounding the peak LOD score; # genes = number of protein coding genes within the support interval; Diff. SNPs = number of differential SNPS predicted by MGI in the interval; SD = value in SD units for the trait. Intervals and positions are in megabases (Mb).

**Table 2:** Differentially expressed genes from C57BL/6J and A/J mice located within the *Xbp1s* and *Socs3* eQTLs

		Gene Name	Entrez ID	Average Expression	*P*	SNP	nsSNP
**Chr 1**	Apcs	*Serum amyloid P-component*	20219	13.12	4.58E-23	4	0
	Darc	*Duffy blood group*, *chemokine receptor*	13349	8.73	1.88E-13	0	0
	Dusp23	*Dual specificity phosphatase 23*	68440	10.02	4.40E-12	38	0
	Ephx	*Epoxide hydrolase 1*, *microsomal*	13849	13.18	1.07E-04	52	0
	F11 r	F11 receptor	16456	9.52	1.03E-18	112	0
	Fcer1g	Fc receptor, IgE, high affinity I,polypeptide	14127	8.53	1.35E-30	39	0
	Fmo3	Flavin containing monooxygenase 3	14262	11.23	2.04E-08	4	0
	Gm5561	*Predicted gene 5561*	433941	11.19	3.03E-12	0	0
	Mgst3	Microsomal glutathione S-transferase 3	66447	11.02	1.60E-03	0	0
	Pex19	Peroxisomal biogenesis factor 19	19298	12.31	7.24E-11	65	1
	Psen2	*Presenilin 2*	19165	10.9	9.43E-18	31	0
	Rgs4	Regulator of G-protein signaling 4	19736	8.88	1.41E-06	25	0
	Srp9	*Signal recognition particle 9*	27058	9.61	1.29E-14	28	0
	Susd4	*Sushi domain containing 4*	96935	9.05	4.36E-05	10	0
	Uap1	UDP-N-acetylglucosamine pyrophosphorylase 1	107652	9.5	1.47E-16	28	0
**Chr 11**	Atox1	ATX1 (antioxidant protein 1) homolog 1 (yeast)	11927	12.24	2.06E-13	0	0
	Dhrs1	Dehydrogenase/reductase (SDR family) member 1	52585	12.1	7.32E-11	0	0
	Igtp	Interferon gamma induced GTPase	16145	11.05	2.70E-07	23	2
	Pemt	Phosphatidylethanolamine N-methyltransferase	18618	13.15	1.72E-09	30	0
	Snap47	Synaptosomal-associated protein, 47	67826	8.89	1.30E-14	6	0
	Timd2	T-cell immunoglobulin and mucin domain containing 2	171284	12.74	1.49E-18	41	0
	Timd4	T-cell immunoglobulin and mucin domain containing 4	276891	8.87	2.88E-08	49	2
	Trim11	Tripartite motif-containing 11	94091	8.32	8.34E-28	30	0
	Zfp39	Zinc finger protein 39	22698	8.44	9.31 E-16	33	2
**Chr 12**	Adssl1	Adenylosuccinate synthetase like 1	111565	11.62	6.94E-13	56	0
	Amn	Amnionless	93835	8.42	1.56E-13	7	1
	AW555464	Expressed sequence AW555464	217882	11.28	1.24E-15	43	2
	Ckb	Creatine kinase, brain	12709	9.81	8.69E-05	7	0
	Ppp1r13b	Protein phosphatase 1, regulatory subunit 13B	21981	8.89	9.16E-13	98	0

Differentially expressed genes in the eQTLs. The genes on Chr 1 and Chr 11 are in both the *Xbp1s* and *Socs3* eQTL. The genes on Chr 12 are in a *Socs3* eQTL. SNP, differential SNPs between strains; nsSNP, nsSNPs differential SNPs.

### Consomic mice replacing A/J Chr 11, but not Chr 1, into C57BL/6J mice reverses the *Xbp1s* and *Socs3* phenotypes

**Figure 4:**
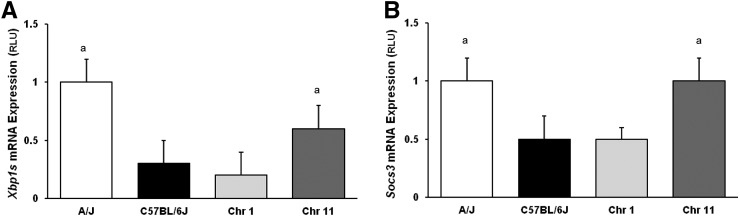
Hepatic mRNA expression of *Xbp1s* and *Socs3* in A/J, C57BL/6J, and C57BL/6J-Chr 1A/J/NaJ and C57BL/6J-Chr 11A/J/NaJ mice. Male A/J, C57BL/6J, C57BL/6J-Chr 1A/J/NaJ, and C57BL/6J-Chr 11A/J/NaJ mice were fed a 60% high-fat, high-caloric (HFHC) diet for 8 wk and hepatic mRNA expression of (A) *Xbp1s* and (B) *Socs3* gene expression measured using RT-PCR. RLU, random light units. Mean ± SEM. A/J (white), C57BL/6J (black), C57BL/6J-Chr 1A/J/NaJ (Chr 1, light gray), and C57BL/6J-Chr 11A/J/NaJ (Chr 11, dark gray). ap < 0.05 *vs.* both C57BL/6J and C57BL/6J-Chr 1A/J/NaJ mice (n = 8).

Because eQTLs for both *Xbp1s* and *Socs3* were identified on chromosomes 1 and 11, we performed RT-PCR of *Xbp1s* and *Socs3* on hepatic mRNA isolated from C57BL/6J-Chr 1^A/J^/ NaJ and C57BL/6J-Chr 11^A/J^/ NaJ CSS mice, and a new cohort of A/J and C57BL/6J mice, fed the HFHC diet for 8 wk. Hepatic gene expression of *Xbp1s* was 1.0 ± 0.2 and 0.6 ± 0.2 in A/J and C57BL/6J-Chr 11^A/J^/ NaJ mice, and 0.3 ± 0.2 and 0.2 ± 0.2 in C57BL/6J and C57BL/6J-Chr 1^A/J^/ NaJ mice (Figure 4). Therefore, *Xbp1s* gene expression was higher in C57BL/6J-Chr 11^A/J^/ NaJ mice than in C57BL6/J mice (*P* < 0.05), but was similar in C57BL/6J-Chr 1^A/J^/ NaJ mice and C57BL/6 mice. Replacement of A/J chromosome 11, but not chromosome 1, into strain C57BL/6 mice reversed the *Xbp1s* phenotype. Hepatic *Socs3* gene expression was similar (1.0 ± 0.2 and 1.0 ± 0.2 RLU) in A/J and C57BL/6J-Chr 11^A/J^/ NaJ mice, but was also lower in both the C57BL/6J and C57BL/6J-Chr 1^A/J^/ NaJ mice, at 0.5 ± 0.2 and 0.5 ± 0.2 RLU, respectively (*P* < 0.05; n = 8). Transfer of A/J chromosome 11, but not chromosome 1, into C57BL/6J mice reversed the *Socs3* phenotype, similar to the findings of hepatic *Xbp1s* mRNA expression.

### Identification of QTL for the metabolic syndrome using F_2_ (A/J × C57BL/6J) mice fed a HFHC diet

Table S2 reveals significant differences that were noted between C57BL/6J mice and A/J mice with respect to several metabolic syndrome phenotypes: body weight gain, hepatic triglycerides, fasting serum insulin, fasting serum glucose, and QUICKI, Therefore, we performed QTL analysis with respect to these phenotypes. QTL analysis identified several chromosomal loci associated with body weight gain, fasting serum insulin, and fasting serum glucose; however, no QTL were identified for the phenotypes of hepatic triglycerides or QUICKI (Table S3).

### Identification of eQTLs for hepatic *Xbp1* and *Socs3* gene expression in the CCl_4_ model

**Figure 5:**
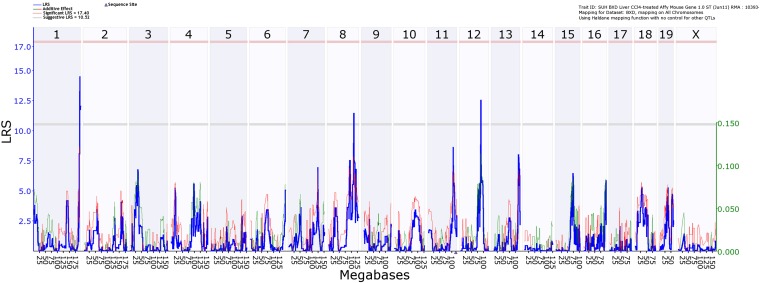
Interval mapping/regression analysis of BXD mice treated with carbon tetrachloride (CCl4). Chow-fed BXD murine lines were treated with CCl4 and *Socs3* hepatic gene expression was measured. eQTLs were identified using interval mapping as implemented in GeneNetwork.

To further confirm that the regulation of hepatic *Xbp1s* and *Socs3* expression is altered in a second animal model of liver injury, we used the carbon tetrachloride (CCl_4_) model of chronic liver injury and fibrogenesis. Wild-type mice fed chow without CCl_4_ do not develop spontaneous hepatic fibrosis. For this analysis, we searched for coinciding regulatory loci within the *Socs3* and *Xbp1* eQTLs on mouse chromosome 1 (164.4–190.5 Mb), chromosome 11 (41.1–73.1 Mb), and chromosome 12 (109.9–117.4 Mb) in the BXD reference population to allow systematic mapping and integration of multiple complex traits (Andreux *et al.* 2012). We initially mapped hepatic expression values of *Socs3* and *Xbp1s* using our microarray-based expression dataset. Figure 5 shows the interval mapping/regression analysis for the phenotype of hepatic *Socs3* expression. The most significant loci were identified on chromosome 1 at 185.2–186.6 Mb for *Xbp1* (LOD 3.5; *P*_G_ = 0.08) and 193.3–197.1 Mb for *Socs3* (LOD 4.4, *P*_G_ = 0.01), chromosome 4 at 62.4–68.9 Mb for *Xbp1* (LOD 3.1, *P*_G_= 0.02), and chromosome 12 at 83.9–89.9 Mb for *Socs3* (LOD 5.6; *P*_G_ = 0.002). Of note, the eQTLs for *Socs3* and *Xbp1* on chromosomes 1 that determine hepatic expression levels after CCl_4_ challenge coincide with loci that determine gene expression on the HFHC diet as well as eQTLs in control BXD. These findings suggest that baseline expression differences due to genetic variation confer susceptibility to liver injury. Furthermore, the expression differences are inversely correlated with Sirius red-stained collagen areas in livers from CCl_4_-treated animals (*P* < 0.001) (Hall *et al.* 2014). We also identified several genes potentially regulated from within the *Socs3* and *Xbp1* eQTLs. Table 3 summarizes these *cis*-regulated differentially expressed genes identified using the BXD lines that co-localize with the eQTLs identified in the HFHC model. Among the regulated genes, only *Darc* and *Zfp39* inherit nsSNPs in coding regions differentiating between C57BL/6J and A/J or DBA/2J mice, respectively.

**Table 3:** Differentially expressed hepatic genes identified in *Xbp1s* and *Socs3* eQTLs regions that are *cis*-regulated in BXD mice treated with CCl_4_

eQTL (BXD)				*Cis*-Regulated Genes (Gene ID)	Gene Location (Mb)	Mean Hepatic mRNA Expression	Max. LOD	Max. LOD Location (Chr:Mb)	nsSNP Variants		
Chr	Trait	Location (Mb)	Allele Effect*^a^*						B	D	A/J
				*Mgst3:* Microsomal glutathione S-transferase 3 (66447)	169.3	9.8	6.7	Chr1: 169.2			
				*Pex19:* Peroxisomal biogenesis factor 19 (19298)	174.1	11.2	2.4	Chr1: 125.0			
**1**	*Socs3*	187.0–187.7	−0.122 (B)	*Dusp23:* Dual specificity phosphatase 23 (68440)	184.6	9.6	5.7	Chr1: 174.2			
	*Xbp1*	186.2–186.6	−0.102 (B)	*Apcs:* Serum amyloid P-component (20219)	174.8	12.0	7.5	Chr1: 173.0			
				*Darc:* Duffy blood group, chemokine receptor (13349)	175.3	8.5	5.4	Chr1: 174.2	A	G	G
				*Psen2:* Presenilin 2 (19165)	182.2	10.8	4.3	Chr1: 182.2			
**11**	*Socs3*	106.5–106.8	−0.097 (B)	*Zfp39:* Zinc finger protein 39 (22698)	58.7	8.7	10.6	Chr11: 58.9	A	G	G
**12**	*Socs3*	88.2–89.9	0.114 (D)	*Amn:* Amnionless (93835)	112.5	8.4	7.1	Chr12: 112.4	T	C	C

a Allele effects are estimated change in trait mean (gene expression) by replacement of one allele by the other: negative values indicate that higher expression values are produced by the C57BL/6J (B) allele and positive values by DBA/2J (D) allele.

## Discussion

With the prevalence of NALFD rapidly increasing, it is essential that we gain a better understanding of the pathogenesis and progression of fatty liver disease. Although NAFLD has a hereditary component, the genetics of NAFLD are poorly understood (Schwimmer *et al.* 2009; Wilfred de Alwis and Day 2008). HFHC diets have been extensively utilized for murine models of obesity, diabetes, and fatty liver. Mice fed this diet develop obesity and many of the components of the metabolic syndrome in a strain-specific manner (Minkina *et al.* 2012; Jiang *et al.* 2005; Becker *et al.* 2004; Schmid *et al.* 2004; Kobayashi *et al.* 2004; Rangnekar *et al.* 2006). In this study, A/J and C57BL/6J mice were fed a HFHC diet for 8 wk to identify eQTLs and candidate genes that contribute to NAFLD.

*Xbp1s* is a key regulator in the IRE1α signaling pathway of the unfolded protein response (UPR), which is activated by ER stress. Hepatic ER stress occurs due to insulin resistance, diabetes, obesity, and several liver diseases, including fatty liver, hepatitis C, and alpha-1 antitrypsin deficiency (Ji 2008; Lawless *et al.* 2004; Li *et al.* 2009; Puri *et al.* 2008; Malhi and Kaufman 2011). In addition to its role in the UPR, hepatic *Xbp1s* is an important regulator of fatty acid synthesis and systemic lipid metabolism (Lee *et al.* 2008; Sha *et al.* 2009; Ye *et al.* 2012). Dysregulation of hepatic *Xbp1s* expression has been shown to be important in the pathogenesis of human NASH (Puri *et al.* 2008). In addition, several studies have also demonstrated the important role of *Socs3* in the pathogenesis of nonalcoholic steatohepatitis, regulating insulin sensitivity, leptin signaling, glucose metabolism, lipogenesis, and fatty liver (Wang *et al.* 2009; Ihle 1995; Muraoka *et al.* 2003; Kievit *et al.* 2006; Howard *et al.* 2004; Dittrich *et al.* 2012; Sachithanandan *et al.* 2010).

When fed a HFHC diet, hepatic gene expression of both *Xbp1s* and *Socs3* was greater in A/J mice compared with C57BL6/J mice. In contrast, there were no differences in hepatic gene expression of either *Xbp1s* or *Socs3* in chow-fed mice, indicating that the phenotypic change is elicited by feeding the HFHC diet for 8 wk. The increased expression of both genes is likely a cellular response to prevent further liver injury, with activation of the unfolded protein response (UPR) and suppression of inflammatory cytokines.

After determining that both hepatic *Xbp1s* and *Socs3* mRNA expression was differentially expressed in HFHC diet-fed A/J and C57BL/6 mice, we applied eQTL analysis to identify genetic loci that may be important in the regulation of these genes that influence the pathogenesis of fatty liver disease. We identified overlapping eQTLs on mouse chromosomes 1 and 11 that were associated with both hepatic *Xbp1s* and *Socs3* expression. We also identified an additional eQTL for *Socs3* on chromosome 12. Of note, the murine genes for *Xbp1* and *Socs3* are located on chromosome 11, although both genes are over 50 Mb away from SNPs rs3684076 and rs3697686 marking the eQTLs. Assuming that a window of ±5 Mb is used to define a *cis*-QTL, this suggests that they are trans eQTLs (http://www.genenetwork.org/gghelp.html). Using microarray analysis, we identified differentially expressed candidate genes in the overlapping eQTL regions on chromosomes 1 and 11; as well as five additional differentially expressed candidate genes in the *Socs3* eQTL on chromosome 12. No genes that are known to be important in the pathogenesis of nonalcoholic steatohepatitis were identified to be differentially expressed in these loci or the syntenic human loci.

Chromosome substitution strains (CSS) of mice are widely used for gene discovery, genetic and epigenetic studies, functional characterizations, and systems analysis (Singer *et al.* 2004; Hines *et al.* 2011).

When C57BL/6J-Chr 11^A/J^/ NaJ mice were fed a HFHC diet for 8 wk, both hepatic *Xbp1s* and *Socs3* gene expression was similar to A/J mice, yet differed from C57BL/6J mice. In contrast, hepatic *Xbp1s* or *Socs3* gene expression in C57BL/6J-Chr 1^A/J^/ NaJ mice remained similar to C57BL/6J mice. This demonstrates that the replacement of the C57BL6/J chromosome 11 with A/J chromosome 11 reverses the gene expression phenotype of both *Xbp1s* and *Socs3* expression from a C57BL/6J to an A/J phenotype, supporting the physiologic relevance of the eQTL. Although C57BL/6J-Chr 1^A/J^/NaJ and C57BL/6J mice had similar hepatic gene expression of *Xbp1s* and *Socs3*, it does not exclude the physiologic relevance of the eQTL on chromosome 1 because replacement of the entire chromosome may also change other nonidentified QTL on the chromosome.

We also identified QTL for the phenotypes of serum insulin levels, serum glucose, and weight gain. A QTL on Chr 1 for the phenotype of serum insulin has a small overlapping region with the eQTLs for *Xbp1s* and *Socs3* on Chr 1. We did not identify any QTLs for the phenotypes of hepatic triglycerides or QUICKI. QTLs for murine hepatic triglyceride have been previously reported (Minkina *et al.* 2012; Lemoine and Serfaty 2012).

C57BL/6J and A/J mice have been commonly used for models of obesity, diabetes, fatty liver, and the metabolic syndrome (Alevizos *et al.* 2007; Hall *et al.* 2010; Li *et al.* 2005; Nishina *et al.* 1993). Several studies have determined that C57BL/6J are sensitive to and that A/J mice are resistant to metabolic syndrome phenotypes, and our findings are similar to published data. However, human nonalcoholic steatohepatitis (fatty liver with inflammation and fibrosis) is associated with inappropriately low levels of hepatic XBP1 expression compared with bland hepatic steatosis (fatty liver without inflammation and fibrosis) (Puri *et al.* 2008). Consistent with these data, we have determined that when liver-specific *Xbp1*-knockout mice in a C57BL/6J background are fed a high-fat/sucrose-fructose diet, they are more susceptible to develop progressive, fibrosing steatohepatitis with less steatosis than flox control mice. Therefore, the hepatic phenotype of the liver-specific *Xbp1*-null mice is similar to that of C57BL/6J mice, whereas flox controls have a phenotype similar as A/J mice. *Socs3* has also been demonstrated in several studies to be an important mediator of both murine and human NASH (Prpic *et al.* 2003; Bergen *et al.* 1999; Parekh *et al.* 1998; Surwit *et al.* 1997; Winzell and Ahren 2004; Livingston *et al.* 1994; Surwit *et al.* 1988; Tilg 2010; Sachithanandan *et al.* 2010; Surwit *et al.* 1995; Watson *et al.* 2000). Previous studies have also identified other QTL for the metabolic syndrome in mice (Jiang *et al.* 2005; Rangnekar *et al.* 2006). In this study, we identified 14 QTL that are related to metabolic syndrome phenotypes, but we did not identify QTL for hepatic triglycerides.

To determine whether the identified regulatory eQTLs for *Xbp1s* and *Socs3* may be important in other forms of liver injury, we used the CCl_4_ model of hepatotoxicity and fibrosis using the BXD lines as an established murine reference panel for systems genetics (Andreux *et al.* 2012). eQTL analysis was performed in F_2_ (A/J × C57BL/6J) mice because feeding a HFHC diet to A/J and C57BL/6J mice is a well-established model for genetic and physiologic studies of the metabolic syndrome. The BXD lines, however, provided an efficient murine model for the experiments administering CCl_4_. In this second model of liver injury, we identified significant regulatory loci for *Socs3* and *Xbp1s* expression on chromosomes 1 and 12, including an overlapping region on chromosome 1 that is influenced by the parental C57BL/6J allele in both experimental crosses.

A GWAS study of the Dallas Heart study population has revealed that a polymorphism of *PNPLA3* (rs738409; I148M) is strongly associated with increased hepatic fat levels and susceptibility to nonalcoholic fatty liver disease (Romeo *et al.* 2008). This polymorphism has subsequently been associated with the severity of NASH, liver stiffness, fibrosis, and potentially hepatocellular carcinoma. Overexpression of the PNPLA3 I148M polymorphism in mice also causes hepatic steatosis and PNPLA3 has been shown to regulate hepatic lipid metabolism (Basantani *et al.* 2011; Chen *et al.* 2010; Li *et al.* 2012; Ruhanen *et al.* 2014; Singal *et al.* 2014; Said 2013; Pingitore *et al.* 2014; Krawczyk *et al.* 2013; Peng *et al.* 2012; Santoro *et al.* 2010; Rotman *et al.* 2010; Speliotes *et al.* 2010; Zain *et al.* 2012). Therefore, increasing evidence indicates that PNPLA3 polymorphisms have an important role in hepatic lipid metabolism and human fatty liver disorders. A TM6SF2 variant confers susceptibility to human nonalcoholic fatty liver disease, as well as influences total cholesterol and myocardial infarction risk (Kozlitina *et al.* 2014; Holmen *et al.* 2014; Glimcher and Lee 2009). Recent data also demonstrate that UDP-galactose-4-epimerase (GalE) is a direct transcriptional target of *Xbp1s* that is located at 136 Mb on chromosome 4 within our QTL interval for weight gain. Several SNPs, including rs2645424, rs343062, SNP rs1227756, rs6591182, rs887304, rs2499604, rs6487679, rs1421201, and rs2710833, have been associated with NAFLD, NASH, or serum aminotransferase elevation (Chalasani *et al.* 2010; Kilpelainen *et al.* 2011). Significant associations with histologic NAFLD have also been identified at variants in or near *NCAN*, *GCKR*, and *LYPLAL1* (Speliotes *et al.* 2011). The roles of these SNPs in fatty liver diseases and lipid metabolism require further investigation.

Hepatic *Xbp1s* is induced by the endoplasmic reticulum stress that occurs in viral hepatitis, alpha-1 antitrypsin deficiency, obesity, insulin resistance, and hepatic steatosis. *Xbp1s* is formed by an atypical splice mechanism in which the endonuclease IRE-12 excises 26 nucleotides from the unspliced *Xbp1* and causes a frame shift that removes an early stop codon. *Socs3* is a negative regulator of cytokine signaling, with expression induced by cytokines such as interferon-γ, interleukin-6, and interleukin-10. None of the candidate genes that we identified are known to regulate *Xbp1s* or *Socs3*, nor have they previously been associated with the pathogenesis of fatty liver.

Human NAFLD is a highly prevalent disease that is closely associated with the metabolic syndrome, although the pathogenesis remains poorly understood. Several studies have shown that genetic factors are important in the pathogenesis of fatty liver; however, many of the specific genetic factors remain unknown. In this study, we have identified overlapping eQTLs for the phenotypes of *Xbp1s* and *Socs3* expression levels, genes that are likely important in the pathogenesis of fatty liver disorders. We have also used microarray analysis to identify differentially expressed candidate genes in the eQTL regions. Future studies using fine-mapping techniques, positional cloning, or genetically altered mice may identify the genes or polymorphisms that are responsible for disease expression. Given the increasing prevalence of NAFLD, it is essential that we further understand the genetics and pathophysiology of fatty liver diseases so that we can develop rationale therapeutic options for this common liver disease.

## Supplementary Material

Supporting Information
