# Supplementary material for: Identification of eQTLs for Hepatic Xbp1s and Socs3 Gene Expression in Mice Fed a High-Fat, High-Caloric Diet
Source: G3 (Bethesda). 2015 Jan 23;5(4):487–96. doi: 10.1534/g3.115.016626 (PMC4390565; doi:10.1534/g3.115.016626)
Supplement: Supporting Information [file supp_5_4_487__index.html]

Identification of eQTLs for Hepatic Xbp1s and Socs3 Gene Expression in Mice Fed a High-Fat, High-Caloric Diet — Supporting Information 

# Identification of eQTLs for Hepatic *Xbp1s* and *Socs3* Gene Expression in Mice Fed a High-Fat, High-Caloric Diet

## Supporting Information for Pasricha *et al.*, 2015

**Files in this Data Supplement:**

- Supporting Information - Tables S1-S3 (PDF, 593 KB)
- Table S1 - Pearson correlation matrix of phenotypes using C57BL/6J and A/J mice. (PDF, 349 KB)
- Table S2 - Phenotypes of A/J, C57BL/6J and F2 (A/J x C57BL/6J) mice. (PDF, 383 KB)
- Table S3 - QTLs for metabolic syndrome phenotypes in studies of C57BL/6J and A/J mice fed a High-Fat, High-Caloric Diet. (PDF, 269 KB)
