# Supplementary material for: Identification of eQTLs for Hepatic Xbp1s and Socs3 Gene Expression in Mice Fed a High-Fat, High-Caloric Diet
Source: G3 (Bethesda). 2015 Jan 23;5(4):487–96. doi: 10.1534/g3.115.016626 (PMC4390565; doi:10.1534/g3.115.016626)
Supplement: Supporting Information [file supp_g3.115.016626_016626SI.pdf]

G3·Genes | Genomes | Genetics

Identification of eQTLs for Hepatic *Xbp1s* and *Socs3* Gene Expression in Mice fed a High-Fat, High Caloric Diet

Sarina Pasricha*[,§], Jane Kenney-Hunt[**], Kristy Anderson*, Nadereh Jafari[§§], Rabea A. Hall***, Frank Lammert***, James Cheverud[§§§], and Richard M. Green*[1]

*Department of Internal Medicine, Northwestern University, Chicago, IL, [§]Department of Internal Medicine, University of North Carolina, Chapel Hill, NC, **Department of Biology and Environmental Science, Westminster College, Fulton, MO, [§§]Center for Genetic Medicine, Northwestern University, Chicago, IL. ***Department of Medicine II, Saarland University Medical Center, Homburg, Germany, [§§§]Department of Biology, Loyola University, Chicago, IL.

Reference Numbers: GEO GSE56987, GeneNetwork GN325;

[1]Corresponding Author: Tarry Building 15-719, 303 East Chicago Avenue, Chicago, IL 60611. E-mail: r-green2@northwestern.edu

DOI: 10.1534/g3.115.016626

Table S1: Pearson correlation matrix of phenotypes using C57BL/6J and A/J mice

	**IL6**	***Socs1***	***Socs3***	***Xbp1s***	**TRI**	**INSULIN**	**GLU**	**QUICKI**	**WT**	**ALT**
**IL6**	1.00									
Socs1	0.50	1.00								
Socs3	0.03	0.11	1.00							
Xbp1s	0.07	0.00	0.75	1.00						
**TRIG**	-0.27	-0.16	0.09	0.11	1.00					
**INSULIN**	-0.19	-0.17	-0.18	-0.19	0.12	1.00				
**GLU**	0.14	0.14	-0.16	-0.17	0.01	0.11	1.00			
**QUICKI**	0.22	0.08	0.09	0.10	-0.18	-0.74	-0.31	1.00		
**WT**	-0.08	-0.06	0.09	-0.15	0.03	0.33	0.02	-0.24	1.00	
**ALT**	-0.02	0.03	0.05	0.06	-0.08	0.16	0.002	-0.14	0.15	1.00

TRI, triglyceride; GLU, glucose; WT, body weight gain

2 S2

Table S2: Phenotypes of A/J, C57BL/6J and F_2 (A/J x C57BL/6J) mice

Phenotype	A/J	C57BL/6J	F_2 (A/J x C57BL/6J)
No. of mice	10	9	265
Weight gain after 8 weeks (g)	7.0 ± 0.3	17 ± 1.2*	16 ± 0.3*
Hepatic TG (mg/dl/g protein)	246 ± 21	667 ± 103*	492 ± 19*†
Fasting serum insulin (uU/mL)	31 ± 3	125 ± 16*	52 ± 3†
Fasting serum glucose (mg/dL)	270 ± 22	624 ± 36*	356 ± 9*†
QUICKI	0.26 ± 0.03	0.21 ± 0.02*	0.25 ± 0.01

Values are means ± SEM, *p < 0.05 compared to A/J mice, †P < 0.05 compared to C57BL/6J mice.

Table S3: QTLs for metabolic syndrome phenotypes in studies of C57BL/6J and A/J mice fed a High-Fat, High-Caloric Diet

Chr	Trait	LOD	SNP	Position (Mb)	Interval (Mb)	# Genes
1	**insulin**	**4.11**	mCV24145570	195	182.1 - 195.0	86
4	**wt gain**	**6.15**	rs3663950	135.7	128.2 - 154.4	431
7	**insulin**	**4.29**	rs4226520	29.8	24.9 - 40.2	297
10	*glucose*	*3.17*	rs13480569	30.7	28.3 - 31.0	15
11	*insulin*	*3.21*	rs13480968	36.5	30.4 - 40.3	47
13	**wt gain**	**6.64**	rs13481871	71.3	57.3 - 80.2	188
13	*glucose*	*3.36*	rs13481983	103.4	97.3 - 112.0	79
14	*insulin*	*3.23*	rs6169105	117.3	106.3 - 122.1	40
15	*glucose*	*2.75*	rs13482673	82.2	72.2 - 101.6	490

Wt gain is body weight gain over 8 weeks. Glucose and insulin represent serum levels. # Genes = the number of genes and predicted genes within the interval. . Genes and physical positions were obtained from the Mouse Genome Informatics Database. QTLs in bold meet the 5% genome-wide significance threshold generated in R/qtl (Broman et al. 2003). QTLs in *italics* meet the less stringent 5% thresholds generated using the method of Li and Ji (Li and Ji 2005; Chen and Storey 2006).
