# Supplementary material for: Identification of eQTLs for Hepatic Xbp1s and Socs3 Gene Expression in Mice Fed a High-Fat, High-Caloric Diet
Source: G3 (Bethesda). 2015 Jan 23;5(4):487–96. doi: 10.1534/g3.115.016626 (PMC4390565; doi:10.1534/g3.115.016626)
Supplement: Supporting Information [file supp_g3.115.016626_TableS1.pdf]

Table S1: Pearson correlation matrix of phenotypes using C57BL/6J and A/J mice

	**IL6**	***Socs1***	***Socs3***	***Xbp1s***	**TRI**	**INSULIN**	**GLU**	**QUICKI**	**WT**	**ALT**
**IL6**	1.00									
Socs1	0.50	1.00								
Socs3	0.03	0.11	1.00							
Xbp1s	0.07	0.00	0.75	1.00						
**TRIG**	-0.27	-0.16	0.09	0.11	1.00					
**INSULIN**	-0.19	-0.17	-0.18	-0.19	0.12	1.00				
**GLU**	0.14	0.14	-0.16	-0.17	0.01	0.11	1.00			
**QUICKI**	0.22	0.08	0.09	0.10	-0.18	-0.74	-0.31	1.00		
**WT**	-0.08	-0.06	0.09	-0.15	0.03	0.33	0.02	-0.24	1.00	
**ALT**	-0.02	0.03	0.05	0.06	-0.08	0.16	0.002	-0.14	0.15	1.00

TRI, triglyceride; GLU, glucose; WT, body weight gain
