# Supplementary material for: Identification of eQTLs for Hepatic Xbp1s and Socs3 Gene Expression in Mice Fed a High-Fat, High-Caloric Diet
Source: G3 (Bethesda). 2015 Jan 23;5(4):487–96. doi: 10.1534/g3.115.016626 (PMC4390565; doi:10.1534/g3.115.016626)
Supplement: Supporting Information [file supp_g3.115.016626_TableS2.pdf]

Table S2: Phenotypes of A/J, C57BL/6J and F_2 (A/J x C57BL/6J) mice

Phenotype	A/J	C57BL/6J	F_2 (A/J x C57BL/6J)
No. of mice	10	9	265
Weight gain after 8 weeks (g)	7.0 ± 0.3	17 ± 1.2*	16 ± 0.3*
Hepatic TG (mg/dl/g protein)	246 ± 21	667 ± 103*	492 ± 19*†
Fasting serum insulin (uU/mL)	31 ± 3	125 ± 16*	52 ± 3†
Fasting serum glucose (mg/dL)	270 ± 22	624 ± 36*	356 ± 9*†
QUICKI	0.26 ± 0.03	0.21 ± 0.02*	0.25 ± 0.01

Values are means ± SEM, *p < 0.05 compared to A/J mice, †P < 0.05 compared to C57BL/6J mice.
