# Supplementary material for: Identification of eQTLs for Hepatic Xbp1s and Socs3 Gene Expression in Mice Fed a High-Fat, High-Caloric Diet
Source: G3 (Bethesda). 2015 Jan 23;5(4):487–96. doi: 10.1534/g3.115.016626 (PMC4390565; doi:10.1534/g3.115.016626)
Supplement: Supporting Information [file supp_g3.115.016626_TableS3.pdf]

Table S3: QTLs for metabolic syndrome phenotypes in studies of C57BL/6J and A/J mice fed a High-Fat, High-Caloric Diet

Chr	Trait	LOD	SNP	Position (Mb)	Interval (Mb)	# Genes
1	**insulin**	**4.11**	mCV24145570	195	182.1 - 195.0	86
4	**wt gain**	**6.15**	rs3663950	135.7	128.2 - 154.4	431
7	**insulin**	**4.29**	rs4226520	29.8	24.9 - 40.2	297
10	*glucose*	*3.17*	rs13480569	30.7	28.3 - 31.0	15
11	*insulin*	*3.21*	rs13480968	36.5	30.4 - 40.3	47
13	**wt gain**	**6.64**	rs13481871	71.3	57.3 - 80.2	188
13	*glucose*	*3.36*	rs13481983	103.4	97.3 - 112.0	79
14	*insulin*	*3.23*	rs6169105	117.3	106.3 - 122.1	40
15	*glucose*	*2.75*	rs13482673	82.2	72.2 - 101.6	490

Wt gain is body weight gain over 8 weeks. Glucose and insulin represent serum levels. # Genes = the number of genes and predicted genes within the interval. . Genes and physical positions were obtained from the Mouse Genome Informatics Database. QTLs in bold meet the 5% genome-wide significance threshold generated in R/qtl (Broman et al. 2003). QTLs in *italics* meet the less stringent 5% thresholds generated using the method of Li and Ji (Li and Ji 2005; Chen and Storey 2006).
